# Performance evaluation of RDT, light microscopy, and PET-PCR for detecting *Plasmodium falciparum* malaria infections in the 2018 Zambia National Malaria Indicator Survey

**DOI:** 10.1186/s12936-021-03917-6

**Published:** 2021-09-28

**Authors:** Mulenga C. Mwenda, Abebe A. Fola, Ilinca I. Ciubotariu, Conceptor Mulube, Brenda Mambwe, Rachael Kasaro, Moonga B. Hawela, Busiku Hamainza, John M. Miller, Giovanna Carpi, Daniel J. Bridges

**Affiliations:** 1 PATH Malaria Control and Elimination Partnership in Africa (MACEPA), National Malaria Elimination Centre, Ministry of Health, Chainama Grounds, Lusaka, Zambia; 2 Department of Biological Sciences, Purdue University, West Lafayette, IN USA; 3 National Malaria Elimination Centre, Ministry of Health, Chainama Hospital and College Grounds, Lusaka, Zambia; 4 Purdue Institute of Inflammation, Immunology and Infectious Disease, Indiana, USA

**Keywords:** Diagnostics, *P. falciparum*, Malaria, Survey, Prevalence, Light microscopy, PCR, RDT

## Abstract

**Background:**

Zambia continues to advance on the path to elimination with significant reductions in malaria morbidity and mortality. Crucial components that have contributed to progress thus far and are necessary for achieving the national malaria elimination goals include properly identifying and treating all malaria cases through accurate diagnosis. This study sought to compare and assess the diagnostic performance of Rapid Diagnostic Tests (RDT) and Light Microscopy (LM) with photo-induced electron transfer polymerase chain reaction (PET-PCR) as the gold standard using 2018 Malaria Indicator Survey (MIS) data across Zambia to better understand diagnostic accuracy metrics and how these vary across a transmission gradient.

**Methods:**

Cross-sectional samples collected in a nationally representative survey from 7 provinces in Zambia were tested for the presence of malaria parasites by light microscopy (LM), rapid diagnostic test (RDT) and the gold standard PET-PCR. Diagnostic performance was assessed including sensitivity, specificity, negative- and positive-predictive values across a wide malaria transmission spectrum. Diagnostic accuracy metrics were measured, and statistically significant differences were calculated between test methods for different outcome variables.

**Results:**

From the individuals included in the MIS, the overall prevalence of *Plasmodium falciparum* malaria was 32.9% by RDT, 19.4% by LM, and 23.2% by PET-PCR. Herein, RDT and LM diagnostic performance was compared against gold standard PET-PCR with LM displaying a higher diagnostic accuracy than RDTs (91.3% vs. 84.6% respectively) across the transmission spectrum in Zambia. However, the performance of both diagnostics was significantly reduced in low parasitaemia samples. Consistent with previous studies, RDT diagnostic accuracy was predominantly affected by a high rate of false positives.

**Conclusions:**

RDTs and LM both perform well across a range of transmission intensities within their respective target applications, i.e., in the community, for the former, where ease of use and speed of result is critical, and at the health facility, for the latter, where accuracy is prioritized. However, the performance of both diagnostic methods is adversely affected by low parasitaemia infections. As Zambia moves towards elimination more sensitive tools may be required to identify the last cases.

**Supplementary Information:**

The online version contains supplementary material available at 10.1186/s12936-021-03917-6.

## Background

Although significant progress has been made globally to reduce malaria burden through a coordinated international effort focused on vector control, accurate diagnosis and appropriate treatment, malaria continues to be a major global public health problem with devastating impacts on human health and livelihood, especially in sub-Saharan Africa [1]. In 2019, there were an estimated 229 million cases and 409,000 deaths recorded from malaria worldwide, with the World Health Organization (WHO) African Region accounting for approximately 94% of cases and deaths [2]. In the past few years, progress has plateaued with respect to reductions in malaria mortality rates and case incidences as put forth by the WHO Global Technical Strategy for Malaria 2016–2030 (GTS). This has raised concerns for continued success in countries on the path to malaria elimination [1, 3]. Moreover, the current COVID-19 pandemic across the globe will likely affect the availability and distribution of key malaria control interventions and thus will impact malaria transmission dynamics landscape [4, 5]. Thus, monitoring malaria prevalence and understanding the changing malaria transmission landscape as interventions are intensified is key to achieving the planned malaria elimination.

To this end, many countries throughout Africa conduct Malaria Indicator Surveys (MIS), i.e. comprehensive and nationally representative household surveys to collect key malaria data [6]. Measured indicators include the use of interventions like long-lasting insecticidal nets (LLINs) or indoor residual spraying (IRS), as well as individual information about malaria diagnosis. These MIS aim to measure intervention implementation in a respective country, monitor malaria and anaemia prevalence, and evaluate the impact of interventions. One country in which the MIS is conducted is Zambia, a malaria endemic country in sub-Saharan Africa, which has made significant progress towards reducing malaria morbidity and mortality over the past decade through integrated malaria control interventions. For instance, national MIS malaria parasite prevalence by microscopy among children under 5 decreased to 9% in 2018 from 17% recorded in 2015 [7, 8]. The Zambia MIS 2018 aimed to assess progress towards achieving the targets of the National Malaria Elimination Strategic Plan 2017–2021, and was conducted by the Zambia Ministry of Health through the National Malaria Elimination Centre (NMEC) with support from partners, e.g. PATH Malaria Control and Elimination Partnership in Africa (MACEPA) and the United States President’s Malaria Initiative [7]. MIS in Zambia are performed every 2 to 3 years at the end of the malaria transmission season during the months of April and May. The 2018 MIS is the sixth MIS carried out in this country since 2006 [7].

In Zambia, the main malaria control interventions include vector control and effective case management [9]. With consistent use of LLINs and IRS, transmission can decline, but this also can lead to increases in spatial heterogeneity of transmission intensity. For example, in Zambia malaria prevalence by light microscopy (LM) ranged from 0.0% in Southern province to 30.4% in Luapula province in 2018 [7]. Therefore, it is of great interest to understand the impact of changes in malaria prevalence on diagnostic performance and consequently case detection, particularly in such regions with heterogeneous transmission.

Currently, Zambia uses two main diagnostic tools for malaria parasite detection: the *P. falciparum* specific histidine-rich protein 2 (HRP2)-based rapid diagnostic tests (RDTs) and light microscopy (LM) [10]. RDTs detect *P. falciparum* trophozoite-derived HRP2, which accounts for 98 % of malaria cases and morbidity in Zambia and the region at large [7, 9, 11]. RDTs are easy to use, do not require specialized training, can be used by any caregiver, e.g. community health workers, and are the main diagnostic tool used in community management of malaria by both passive and active case detection [12–14]. In contrast, LM can identify and quantify the presence of multiple malaria species, but requires trained microscopists and additional infrastructure, e.g. staining facilities, microscopes, and electricity [15, 16]. As a result, LM is difficult to implement in a rural setting. Finally, it is worth noting that WHO guidelines suggest at least 100 fields of view need to be examined before declaring a slide negative. In low prevalence and pre-elimination settings where there are very few infections to identify this will translate to very low throughput.

Molecular methods can detect parasitaemias below LM and RDTs limit of detection (LOD), making them excellent gold standards to assess diagnostic performance [17–19]. Like LM, multiple *Plasmodium* species can be simultaneously identified with PET-PCR, and in areas of low transmission that are approaching elimination, these other malaria species may become more relevant [17, 20, 21].

As Zambia continues to progress toward malaria elimination, it is essential to diagnose all malaria cases accurately to enable appropriate treatment. Understanding how RDTs, LM, and PET-PCR diagnostics compare across the Zambian transmission gradient will be crucial to achieving this goal. This study, therefore, aimed to assess the diagnostic performance of RDTs and LM for diagnosis of *P*. *falciparum* malaria using PET-PCR as the gold standard. Understanding how these three different diagnostics perform and quantifying diagnostic accuracy will provide insights on prevalence of malaria infection and how these metrics vary according to transmission intensity.

## Methods

### Study design

**Fig. 1:**
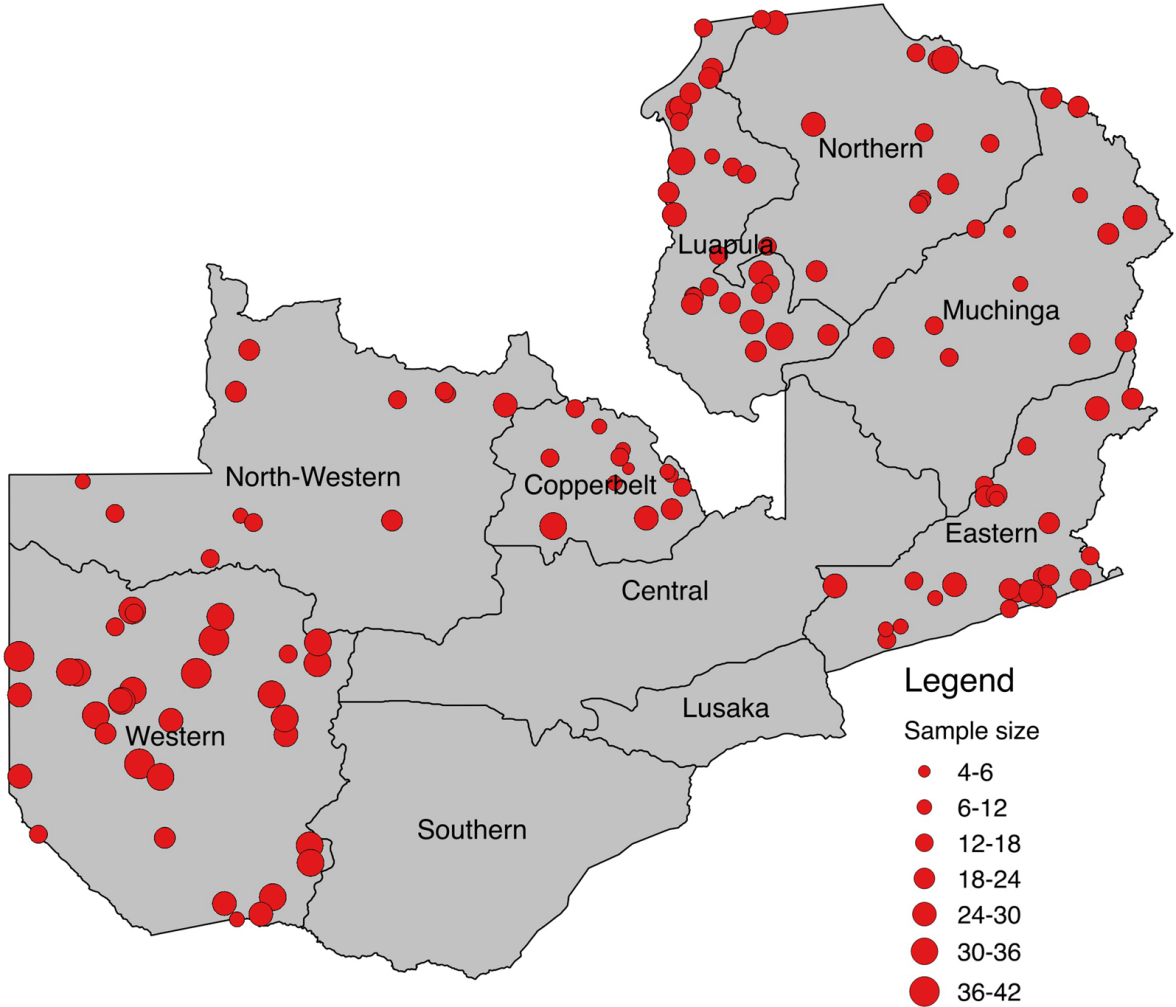
Map of Zambia showing 3153 DBS samples across seven provinces included in the diagnostic method comparison analysis. Sample collection locations or clusters (red circles) are shown proportional to sample size

In 2018, a MIS was performed that randomly surveyed a total of 4177 households across 179 standard enumeration areas from ten provinces in Zambia [7], testing all children under the age of 5 (except Western province where children up to 9 years old were included) with an RDT (SD Bioline Malaria Ag Pf, Standard Diagnostics Inc., Republic of Korea) and collecting a thick smear microscopy slide and dried blood spot (DBS). The survey used a nationally representative two-stage stratified clustering sampling strategy to get country representative samples for the accurate estimation of malaria prevalence and other indicators across Zambia. High transmission provinces such as Luapula and Western were oversampled to better capture the wide range of prevalence. The MIS was conducted from mid-April to late May 2018, which coincides with the end of the malaria transmission season. Provinces with very low PET-PCR positivity, namely Central province with 0.46% positivity (1/213) and Lusaka province with 1.04% positivity (2/193), or zero positives (Southern province) were excluded to avoid sampling bias and reduced precision in downstream diagnostic metrics calculation which used PET-PCR as gold standard. Moreover, samples missing PET-PCR, RDT, and LM results were excluded from the final data analysis. A total of 3153 DBS samples fulfilled the inclusion criteria from seven provinces across Zambia (Fig. 1).

### Laboratory methods

#### Sample and data collection

Before performing the MIS, training was provided for health workers on how to perform an RDT (SD Bioline Malaria Ag Pf, Standard Diagnostics Inc., Republic of Korea), prepare a DBS and thick smear slide as well as how to interview community members and collect survey responses [7]. RDT testing was performed according to manufacturer recommendations. Slides, DBS, used RDTs and data were then transferred to the NMEC in Lusaka for storage and analysis.

#### Microscopy

Thick smear microscopy slides were stained in 10% Giemsa at the NMEC laboratory and read independently by two malaria microscopy-competent technicians. Arbitration of discordant results was performed by a third reader. A blood smear was considered negative when no parasites were detectable after examining 100 high power fields. For positive smears, parasites were counted in reference to 200 white blood cells (WBC) when *Plasmodium* parasites were more than 10, or 500 WBC when *Plasmodium* parasites were less than 10, and then converted into a parasite concentration [22].

#### DNA extraction

DNA was extracted from a single 6-mm punch (equivalent to roughly 13 µL of blood) from each DBS using the QIAamp DNA mini kit (QIAGEN, Hilden, Germany) and eluted in 100 µL of Elution buffer. Punchers were cleaned by making 5 blank punches on clean filter paper between samples. A subset of blank punches was extracted for testing to ensure that there was no cross-over between samples. Extracted DNA was stored at 4 °C for immediate analysis or at − 20 °C for longer term storage.

#### PCR detection

Extracted parasite DNA was detected by real-time PET-PCR, on a LightCycler 480 (Roche, Basel, Switzerland). In brief, all samples were tested in duplicate (5 µL of template, equivalent to 0.7 µL of whole blood) in a duplex reaction with *Plasmodium* spp. and *P. falciparum* primers labelled with FAM and HEX fluorophores, respectively, as previously described [20, 23, 24]. Samples were scored positive when both replicates had cycle threshold values of < 40 and standard deviation of < 2. A limiting dilution series of 3D7 *P. falciparum* genomic DNA (MRA-151G, ATCC, Manassas, VA), obtained through BEI Resources, National Institute of Allergy and Infectious Diseases, National Institutes of Health, contributed by David Walliker, of a known parasitaemia was assayed three times in duplicate by PET-PCR. The standard curves generated from this series established a comparable LOD, as previously published [9], and were used to determine parasitaemia.

### Statistical analysis

Number of DBS samples per province were counted and compiled using dplyr and tidyverse function R package [25, 26]. Using PET-PCR as the ‘gold standard’, diagnostic metrics (Sensitivity, Specificity and Predictive values) were calculated using the epi.test function (‘epiR’ version 1.0–15 R package) [27]. Figure visualization and statistical analyses (Mann-Whitney U test was used to measure differences among two groups) were performed using GraphPad Prism (version 7.0.1 for Mac, GraphPad Software, San Diego, California, USA, www.graphpad.com) and a *P* value of ≤ 0.05 was considered statistically significant.

## Results

### Descriptive statistics

**Fig. 2:**
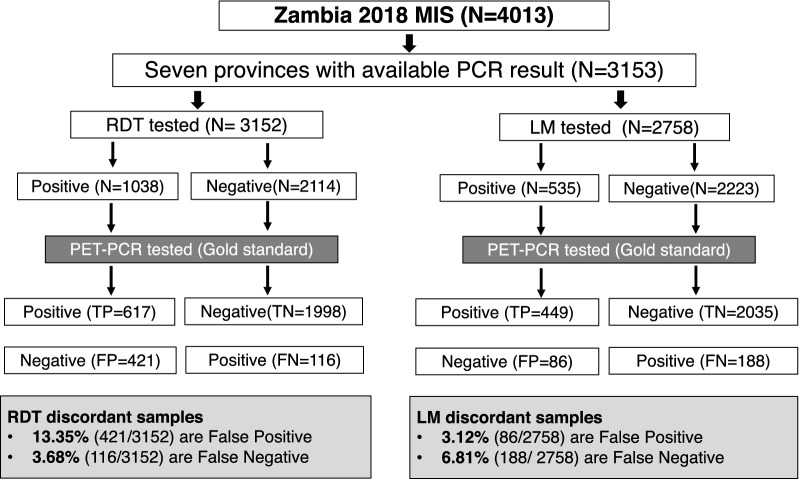
Schematic flow chart showing study participant and diagnostic tests discordance. PET-PCR = Photo-Induced Electron Transfer Polymer Chain Reaction, RDT = Rapid Diagnostic Test, LM = Light Microscopy, TP = True Positive, TN = True Negative, FP = False positive, FN = False Negative

Overall, 32.9% (1038 /3152), 19.4% (535/2758), and 23.2% (733/3153) of study participants were positive for *P. falciparum* by RDT, LM, and PET-PCR respectively across seven provinces. 17.0% (537/3152) of RDT results and 9.9% (274/2758) of LM results were discordant (reported as either falsely positive or falsely negative) when compared to PET-PCR (Fig. 2). RDTs identified more false positives, while LM identified more false negatives (FN), although the overall number of FN samples was relatively low. Note that the variation RDT vs. LM total results was due to the large number of samples that were not tested by LM and thus excluded from the final data analysis.

**Fig. 3:**
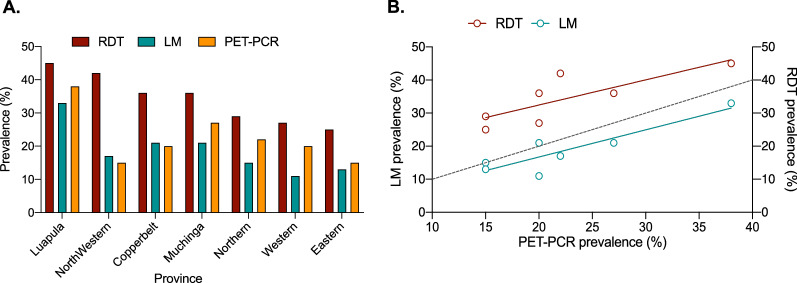
*Plasmodium falciparum* malaria prevalence and its comparison across provinces by the three diagnostic methods. **A** Prevalence of *P. falciparum* malaria per province for each diagnostic assay, ranked in decreasing order of RDT prevalence. **B** Correlation between RDT and LM prevalence against PET-PCR prevalence. Dashed line shows where x = y, i.e. perfect prevalence correlation between the PCR and LM or RDT diagnostics

Diagnostic prevalence per province was highly heterogenous as expected given the transmission gradient across Zambia. Specifically, RDT prevalence ranged from 25.4% to 44.7%, LM from 11.3% to 32.6%, and PET-PCR from 15.4% to 38.0% (Fig. 3A). Overall, however, there was a positive correlation between infection prevalence at the provincial level (Fig. 3B) across the three methods indicating agreement between these three diagnostic methods to estimate *P. falciparum* malaria prevalence and burden across the examined transmission gradient in Zambia.

### Diagnostic performance

Diagnostic sensitivity, (the probability that a test result will be positive when the disease is present) and specificity (the probability that a test result will be negative when the disease is not present) for RDTs showed higher sensitivity 84% (95% CI = 81–87), but lower specificity 83% (95% CI = 81–84) when compared to LM at 71% (95% CI = 67–74), and 96% (95% CI = 95–97) respectively. Positive Predictive Value (PPV) (the probability that the disease is present when the test is positive) and Negative Predictive Value (NPV) (the probability that the disease is not present when the test is negative) were also measured and showed that RDTs had very low PPV 59% (95% CI = 56–62) compared to LM at 84% (95% CI = 81–87) (Table 1).

**Table 1:** Comparison of RDT and LM diagnostic metrics across Zambia

Diagnostic	Result	Positive	Negative	Total	Sensitivity (95% CI)	Specificity (95% CI)	PPV (95% CI)	NPV (95% CI)
RDT	Positive	617	421	1038	84%	83%	59%	95%
	Negative	116	1998	2114				
	Total	733	2419	3152	(81–87)	(81–84)	(56–62)	(93–95)
Microscopy	Positive	449	86	535	70	96	84	92
	Negative	188	2035	2223				
	Total	637	2121	2758	(67–74)	(95–97)	(81–87)	(91–93)

PPV: positive predictive value; NPV: negative predictive value

Diagnostic accuracy, calculated as the proportion of true positive and true negatives among all individuals tested, was consistently higher for LM (91.3 %) vs. RDT (84.6 %) across all seven provinces. Furthermore, there was no obvious variation in diagnostic accuracy with prevalence.

**Fig. 4:**
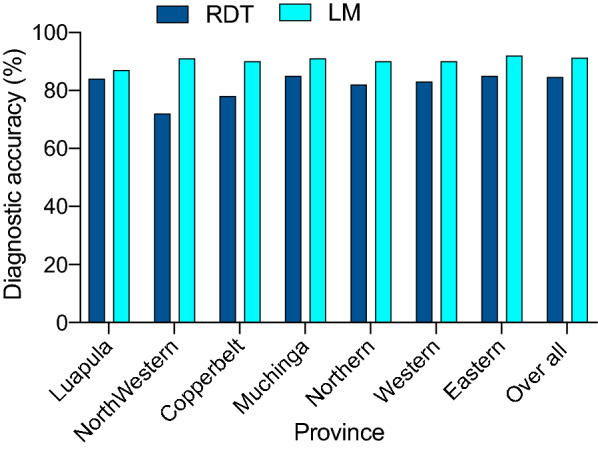
Diagnostic performance metrics of RDTs (**A**) and LM (**B**) across Zambia, using PET-PCR as the gold standard. PPV- positive predictive value, NPV - negative predictive value

Other metrics found that LM had a statistically significantly higher specificity (range = 94–98 %) compared to that of RDT (range = 68–85%). In contrast, RDT showed high sensitivity (range = 76–96 %) but very low PPV (range = 36–75 %) compared to the sensitivity of LM (range = 54–78%), PPV (range = 71–90%) (Fig. 4). For both diagnostics, while specificity and NPV remained similar across the prevalence range, both PPV and sensitivity appeared to decline as prevalence reduced (Fig. 4).

### Effect of parasite density

**Fig. 5:**
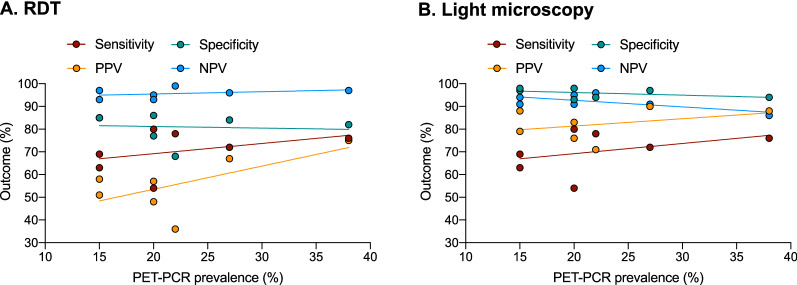
Comparison of parasitaemia for RDT (**A**) and LM (**B**) samples stratified by diagnostic result. A statistically significant difference in parasitaemia level between RDT/LM negative vs. RDT/LM positive samples was identified. ** = p < 0.001

A total of 116 false negatives i.e., PET-PCR positive/RDT negative were identified. The parasitaemia in these samples (median = 7.31 parasites/µL) was significantly lower (P < 0.001) than the parasitaemia (median = 106.1 parasites/µL) in true positive (TP) samples, i.e., PET-PCR positive/RDT positive (Fig. 5A). A similar difference was observed with the 188 LM false negatives, whose parasitaemia (median = 5.65 parasites/µL) was significantly lower (P < 0.001) than the parasitaemia in LM true positives (median = 197 parasites/µL) (Fig. 5B). Correlation analysis between diagnostic metrics and parasite prevalence revealed a positive statistically significant correlation for both RDT PPV (r = 0.85, *P. *value = 0.009) and LM PPV (r = 0.75, *P. value* = 0.021), respectively. No other statistically significant correlations between other diagnostic metrics (Sensitivity, Specificity and NPV) and parasite prevalence were identified.

### Persistent antigenaemia

To assess whether individuals may have had a recent malaria infection, self-reported fever or taking drugs for a fever in the past 2 weeks was used as a proxy. In this group of individuals, the RDT false positive rate of 28.2% (140/496), was almost double that of those not reporting recent fever at 14.6% (281/1923).

## Discussion

Malaria was placed at the forefront of the global public health agenda in 2000 when the Roll Back Malaria Partnership announced a commitment to halving malaria cases by 2015 [28]. A decade later, a 21% reduction in the malaria burden (clinical cases) had been achieved, with elimination now being pursued [1, 28, 29]. Underpinning these aims is the need for early, accurate diagnosis and effective treatment of infections [30], to reduce onward transmission and prevent the development of severe malaria [1]. Ensuring that diagnostics continue to perform well, through regular assessments in all transmission settings, is critical for successful disease management, surveillance, and reaching targets such as elimination [31]. The detection of *Plasmodium* antigens using RDTs is widely used for malaria diagnosis. RDTs are inexpensive, do not require advanced sample preparation or specialized training, and rapidly generate results [13]. This combination has transformed malaria case management from an emphasis on symptomatic presumptive diagnosis to one focused on diagnostic confirmation [3]. RDTs are now also used in active case detection (ACD) to identify infections in the community both as an intervention and as a means to assess changes in prevalence [32]. However, the sensitivity and specificity of RDTs varies significantly between commercial providers [33–35]. RDT and LM diagnostic performance was therefore compared against PET-PCR as a gold standard across a transmission gradient in Zambia with samples collected in a nationally representative MIS in 2018. Considering that each of these diagnostics are fundamentally different in their approach, a certain level of discordance is expected. For example, RDTs directly detect the presence of a parasite protein, while PCR amplifies parasite DNA to give increased sensitivity. Depending on protein/DNA stability and target, a positive RDT/PCR result may or may not be associated with intact parasites. In contrast, LM directly visualizes the parasite, but is dependent on the skill of the microscopist for accurate identification.

While both diagnostics performed well, overall LM had a higher diagnostic accuracy (91.3%) than RDTs (84.6%), supporting the historical use of LM as the gold standard diagnostic [1]. It is worth noting that LM specificity and PPV (Table 1), was lower than expected, and it is most likely due to microscopy errors or artifacts, such as Giemsa stain precipitation that mimic parasite ring stage chromatic dots. The discrepancy between LM and RDT accuracy appeared to be predominantly due to increased RDT false positives. This tendency to over-estimate malaria prevalence can be seen in Fig. 3b where RDT prevalence is consistently higher (~ 10%) than PET-PCR prevalence, as well as in the low RDT PPV (range = 36–75%) compared to LM PPV (range = 71–90%). It is unclear from this study whether this relationship would hold below 15% true malaria prevalence, but it seems unlikely that in near elimination settings there would be an ~ 10 % overestimate. This over-estimation has been documented in other studies [36], and is most likely due to persistent antigenaemia post-infection, or to a lesser extent cross reactivity with other infections or autoantibodies [37, 38]. Indeed, the elevated false positive rate in individuals reporting recent fever suggests that this group of individuals had a higher prevalence of malaria infection that either resolved by itself or was cleared with an anti-malarial drug prior to the MIS, both of which would lead to a false positive outcome. In the context of the MIS this is an important finding and while not desirable, unnecessarily treating an additional 10% of the population is arguably better than missing 10 % of the infected population. While this would translate to the consumption of additional treatments, current anti-malarials have excellent safety profiles so is unlikely to unduly affect the cost-benefit analysis. Furthermore, if treatments are long-lived they could act as a prophylaxis. More worryingly, is the chance of false negative diagnostic results. In this case, an infection and, therefore, the chance to break the chain of transmission, is missed. Both diagnostics perform well in this regard (Fig. 2), but RDTs have a noticeably lower FN rate (3.68%) compared to LM (6.81%). Similarly, LM prevalence was consistently below the PET-PCR prevalence suggesting that this diagnostic is more likely to miss infections (Fig. 3b).

**Fig. 6:**
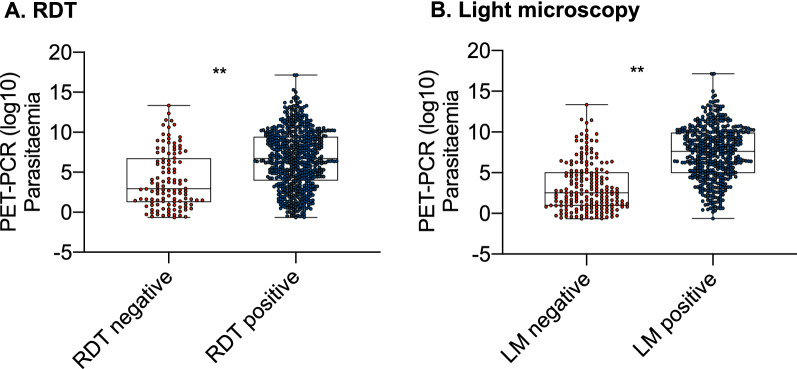
Comparison of diagnostic accuracy between RDT and LM for each province in Zambia. The diagnostic accuracy is the proportion of individuals who are true positive and true negative among all individuals tested

A key question this study set out to address was how diagnostic performance varies across a wide transmission/prevalence range, and in the main, the performance was consistent (Fig. 6). However, it does appear that sensitivity and PPV reduces as prevalence decreases, and more markedly with RDTs vs. LM. This may be due to the diagnostics inherent LOD, i.e., the concentration of the target analyte that must be present for a diagnostic to be able to detect it. For symptomatic infections, that are generally characterized by high parasitaemias, a high LOD is unlikely to affect diagnostic performance. However, in ACD where asymptomatic individuals with very low-density infections are likely to be encountered, the LOD could be critical in determining the success or failure of ACD as an intervention. As expected, the sensitivity of both LM and RDTs reduced as parasitaemia decreased (Fig. 5) suggesting that in settings where there is a high proportion of infections below the diagnostics LOD, e.g. pre-elimination, more sensitive diagnostic tools will be required to find the last asymptomatic infections [39, 40].

Overall, the findings from this study suggest that to more accurately monitor malaria transmission dynamics, countries in pre-elimination settings require more sensitive diagnostic tools to detect asymptomatic and low parasitaemia cases. Molecular techniques, such as nested polymerase chain reaction, quantitative real-time PCR, and ligase detection reaction fluorescent microsphere assay have been developed and have shown greater sensitivity for a broad range of parasitaemias [41, 42]. However, few molecular methods are commonly used in malaria endemic countries as they require a healthy capital budget, advanced laboratories, and skilled workforce. There have been many attempts to develop simplified molecular tools for malaria diagnosis appropriate for low-resource countries [43], e.g. loop-mediated isothermal amplification (LAMP) is a molecular point-of-care test with high specificity and sensitivity (5 parasites/µl of blood) well below the LOD for LM/RDT [44, 45]. Serological diagnostic assays (serosurveillance tools) that detect active or latent infection as well as past exposure may help to assess the malaria burden at a community level more accurately especially in low transmission settings where infections are rare, and surveys, therefore, require large sample sizes to confidently calculate prevalence [46–48].

This study had a number of limitations. Firstly, the MIS sampling design limited enrollment to children preventing the evaluation of diagnostic performance outside this age group. Moreover, there was a marked variation in sample size per province with oversampling from some high transmission areas and under sampling in low transmission provinces, which thus were excluded from this analysis. This study also was not able to address treatment status of study subjects and other non-febrile illnesses among children to assess longevity of *Plasmodium* antigens after treatment and contribution of non-malaria illness on RDT false positivity.

## Conclusions

This *P. falciparum* malaria diagnostic performance evaluation within the nationwide 2018 Zambia Malaria Indicator Survey supports the utility of malaria RDTs for community screening based on their low rate of false negatives and their ease of implementation. In contrast, LM remains the diagnostic method of choice for confirmation of active infections, and accurately treating patients. Notably, the parasitaemia of an infection is one of the main determining factors impacting diagnostic performance and, therefore, accurate determination of malaria parasite prevalence. These findings further suggest that to improve malaria prevalence estimates and case detection in low transmission settings, more sensitive and rapid molecular diagnostic tools to detect asymptomatic and low parasitaemia infections may be necessary to achieve and sustain malaria elimination.

## Supplementary Information

**Additional file 1: Table S1.** Comparison of RDT and LM diagnostic metrics per province in Zambia.

## Data Availability

Raw metadata sets used and/or analysed during the current study are available from the corresponding author on reasonable request and with permission from the NMEC (Additional file 1).

## Abbreviations

ACD: Active case detection
DBS: Dried blood spot
FN: False negative
FP: False positive
HRP2: Histidine Rich Protein 2
IRS: Indoor residual spraying
LM: Light microscopy
LLIN: Long-Lasting Insecticidal Nets
MIS: Malaria Indicator Survey
NMEC: National Malaria Elimination Centre
NPV: Negative Predictive Value
PET-PCR: Photo-induced Electron Transfer Polymerase Chain Reaction
PPV: Positive Predictive Value
RDT: Rapid Diagnostic Test
TN: True Negative
TP: True Positive
WBC: White Blood Cell
WHO: World Health Organization
