# Supplementary material for: Performance evaluation of RDT, light microscopy, and PET-PCR for detecting Plasmodium falciparum malaria infections in the 2018 Zambia National Malaria Indicator Survey
Source: Malar J. 2021 Sep 28;20:386. doi: 10.1186/s12936-021-03917-6 (PMC8477358; doi:10.1186/s12936-021-03917-6)
Supplement: Supplementary file 1 — Additional file 1: Table S1. Comparison of RDT and LM diagnostic metrics per province in Zambia. [file 12936_2021_3917_MOESM1_ESM.docx]

**Table S1.** Comparison of RDT and LM diagnostic metrics per province in Zambia.

| **Diagnostic** | **Metric** | **Copperbelt** | **Eastern** | **Luapula** | **Muchinga** | **Northern** | **North-Western** | **Western** |
| --- | --- | --- | --- | --- | --- | --- | --- | --- |
| **RDT** | Sensitivity (95% CI) | 84.7%  (71.1-93.6) | 86.1%  (76.2-92.5) | 88.3  (83.7-92.1) | 90.2%  (79.8-96.3) | 77.1%  (64.5-86.8) | 97.3%  (85.8-99.2) | 75.6%  (68.7-81.5) |
|  | Specificity (95% CI) | 76.6%  (69.8-82.5) | 85.4%  (81.9-88.4) | 82.1%  (77.9-85.6) | 84.1%  (77.6-89.9) | 84.6%  (79.2-89.2) | 67.8%  (60.9-74.2) | 85.6%  (82.8-88.2) |
|  | PPV  (95% CI) | 47.5%  (36.4-58.8) | 51.4%  (42.9-59.7) | 75.1%  (69.7-79.9) | 67.2%  (55.8-77.1) | 58.2%  (46.5-68.9) | 35.6%  (26.3-45.7) | 57.2%  (50.9-63.4) |
|  | NPV  (95% CI) | 95.2%  (90.4-98.1) | 97.1%  (95.2-98.5) | 91.8%  (88.6-94.5) | 95.8%  (91.3-98.4) | 93.1%  (88.5-96.2) | 99.3%  (96.1-99.6) | 93.2%  (91.1-94.9) |
| **Microscopy** | Sensitivity (95% CI) | 80.4%  (66.1-90.6) | 68.6%  (57.6-78.2) | 75.6%  (70.1-81.2) | 72.2%  (59.2-82.8) | 63.3%  (49.9-75.4) | 78.3%  (61.7-90.2) | 54.2%  (43.7-64.2) |
|  | Specificity (95% CI) | 93.4%  (88.8-96.5) | 96.6%  (94.5-98.1) | 93.8%  (91.2-95.8) | 97.1%  (93.2-99.1) | 97.7%  (94.7-99.3) | 94.1%  (89.8-96.8) | 97.6%  (95.8-98.8) |
|  | PPV  (95% CI) | 75.5%  (61.1-86.6) | 78.7%  (67.6-87.3) | 88.3%  (83.1-92.3) | 89.7%  (77.8-96.6) | 88.4%  (74.9-96.2) | 70.7%  (54.4-83.6) | 82.8%  (71.3-91.2) |
|  | NPV  (95% CI) | 95.1%  (90.7-97.7) | 94.4%  (92.1-96.3) | 86.4%  (82.8-89.4) | 90.5%  (85.2-94.3) | 90.7%  (86.3-94.1) | 95.8%  (92.2-98.2) | 91.1%  (88.2-93.3) |
